# Local Diagnostic Reference Levels for Full-Field Digital Mammography and Digital Breast Tomosynthesis in a Tertiary Hospital in Malaysia

**DOI:** 10.3390/healthcare10101917

**Published:** 2022-09-30

**Authors:** Norhashimah Mohd Norsuddin, Sharveeni Segar, Rathieswari Ravintaran, Norhayati Mohd Zain, Muhammad Khalis Abdul Karim

**Affiliations:** 1 Center for Diagnostic, Therapeutic and Investigative Studies (CODTIS), Faculty of Health Sciences, University Kebangsaan Malaysia, Kuala Lumpur 56000, Malaysia; 2 Medical Imaging Department, School of Health Sciences, KPJ Healthcare University College, Lot PT 17010, Persiaran Seriemas, Kota Seriemas, Nilai 71800, Malaysia; 3 Department of Physics, Faculty of Science, University Putra Malaysia, Serdang 43400, Malaysia edu.my

**Keywords:** digital breast tomosynthesis, full-field digital mammography, diagnostic reference level, average glandular dose, compressed breast thickness

## Abstract

A set of national diagnostic reference levels (DRLs) was established in Malaysia for a range of breast thicknesses in 2013, but no updates for full-field digital mammography (FFDM) and digital breast tomosynthesis (DBT). Due to the increasing number of DBTs used and concern over radiation exposure, this study aimed to explore and establish local diagnostic reference levels for FFDM and DBT in Malaysia health facilities at different compressed breast thickness (CBT) ranges. The CBT, kilovoltage peak (kVp), Entrance surface dose (ESD), and average glandular dose (AGD) were retrospectively extracted from the mammography Digital Imaging and Communications in Medicine (DICOM) header. The 75th and 95th percentile values were obtained for the AGD distribution of each mammography projection for three sets of CBT range. The difference in AGD values between FFDM and DBT at three CBT ranges was determined. The DRLs for FFDM were 1.13 mGy, 1.52 mGy, and 2.87 mGy, while DBT were 1.18 mGy, 1.88 mGy, and 2.78 mGy at CBT ranges of 20–39 mm, 40–59 mm, and 60–99 mm, respectively. The AGD of DBT was significantly higher than FFDM for both mammographic views (*p* < 0.005). All three CBT groups showed a significant difference in AGD values for FFDM and DBT (*p* < 0.005). The local DRLs from this study were lower than the national DRLs, with the AGD of FFDM significantly lower than DBT.

## 1. Introduction

With the addition of new technologies such as Full-field Digital Mammography (FFDM) and Digital Breast Tomosynthesis (DBT), mammography has become the ultimate tool for the early detection of breast cancer. The use of FFDM has resulted in a considerable improvement in the detection of breast cancer when compared with SFM [1]. However, the main drawback of FFDM is its inability to identify abnormalities in dense breast tissue accurately. Dense breast tissue obscures 41% of breast lesions in high-density breast images [2]. So, not all malignancies are captured by FFDM, as some lesions might be undetected because of high breast tissue density [3]. The introduction of Digital Breast Tomosynthesis (DBT) in 2011 has shown some promising evidence in overcoming the limitations of FFDM.

DBT is, in turn, a 3-dimensional (3D) digital mammography. It takes a series of dual-dimensional (2D) low-dose x-ray images from different angles and uses them to synthesize 3D images of the breast [4]. DBT may quickly identify hidden lesions in dense breast tissues, making the image quality superior to SFM and FFDM images [4,5]. In Malaysia, the first DBT modality was installed in 2012. The National Cancer Society of Malaysia is the first health care center to install a Hologic Dimension model, including DBT, in Southeast Asia [6]. The installation of DBT shows Malaysia is on track to catch up with western countries in mammographic technology advancement.

The performance of mammography has increased dramatically from SFM to FFDM and DBT, yet the dose provided by each mammographic technology has not remained constant. Gennaro et al. (2018) showed that the average increase in the dose of DBT compared to FFDM was 38% [7]. While the study by Asbeutah et al. showed that the AGD for a single-view DBT 45–50% was lower than the two-view FFDM technique [8]. However, Gilbert et al. concluded that adding DBT to FFDM doubles the radiation dose a woman would receive in routine breast screening [9]. The variations in the dose when using different technologies in mammography are inevitable. These variations in dose should not be disregarded as they involve breast tissue with a tissue weighting factor of 0.12. It is susceptible to radiation. Also, a high radiation dose increases the high radiation risk to the patient, such as radiation-induced breast cancer [10]. The variations in dose are mainly due to the mammographic technique and the population’s compressed breast thickness [11]. Thus, optimization is needed in regulating the variations in mammographic dose.

In 1996, the International Commission on Radiological Protection (ICRP) introduced the Diagnostic Reference Level (DRL) to optimize the dose for all ionizing radiation [5]. DRL is an optimum range of doses that is safer for patients to undergo examination while not losing the diagnostic value of the image. DRL must be established for each imaging modality to help detect the unusual level of doses given to patients [12]. The DRL is derived from calculating the median value (75th or 95th percentile) of the distribution of Mean Glandular Dose (MGD) measurements from the observed sample. According to the Australian Radiation Protection and Nuclear Safety Agency (ARPANSA), local DRLs should be created within institutions. They must be by the national DRLs if they exist [13]. The ICRP recommends that DRLs for mammography must be updated at regular intervals of three to five years [5]. As per current data, DRLs vary across countries. The DRLs established in Belgium and Japan for FFDM are 2.46 mGy and 2.0 mGy, respectively [12,14]. In Ireland, respectively, 1.75 mGy and 2.6 mGy were established as DRL for FFDM and DBT [15,16]. Although a set of national DRLs was established in Malaysia for a range of breast thicknesses in 2013 [17], no separate DRLs have been reported and established for FFDM and DBT. Since the use of FFDM and DBT has gradually replaced SFM in Malaysia health facilities, and while waiting for national DRLs to be updated, local DRLs should be established at an institutional level, as suggested by the ICRP and International Atomic Energy Agency (IAEA). Therefore, this study aimed to establish the local DRLs for FFDM and DBT in an institution in Malaysia to guide current mammography practice.

## 2. Materials and Methods

Ethics approval was obtained from the National Medical Research Register (NMRR-19-3761-51394) in compliance with the current Ministry of Health (MOH) and National Institutes of Health (NIH) guidelines for conducting research.

This research was conducted retrospectively and carried out in the Department of Radiology of Hospital Kuala Lumpur (HKL), located in Kuala Lumpur, Malaysia’s capital city. This institution was chosen because it has a functional mammography machine at the time of data collection and has a high population density. This institution has a Hologic Selenia Dimension (Hologic Inc., USA) mammographic machine, which has been used since 2006. The image detector of this model uses direct-capture amorphous selenium technology.

The study retrospectively gathered data from 188 patients for FFDM and 333 patients for DBT. Both screening and symptomatic patients who underwent mammographic examination HKL from January 2018 to June 2020 were selected for this study. Data extracted from the mammography Digital Imaging and Communications in Medicine (DICOM) header; AGD in mGy, compressed breast thickness (CBT) in millimeter (mm), age of patients, entrance surface dose (ESD) in mili Gray (mGy), kilovoltage peak (kVp) and milliampere-seconds (mAs). Automatic Exposure Control (AEC) was used to select kVp and mAs for mammography examinations. The four-view mammography images were left craniocaudal (LCC), right craniocaudal (RCC), left mediolateral oblique (LMLO), and right mediolateral oblique (RMLO). The auto filter mode was used. The Hologic vendor utilizes the Boone method to estimate the organ dose that automatically displays after each exposure [18].

Additional views were excluded from the study for patients with breast implants, mastectomy, incomplete data, and less than the standard four mammographic projections. The final data set consisted of 87 and 223 images for each view (RCC, LCC, RMLO, and LMLO) in FFDM and DBT, respectively.

In this study, the Mean Glandular Dose (MGD) or Average Glandular Dose (AGD) for each acquired image was calculated automatically in the mammographic machine and recorded in the system using the methods described by Dance et al. [19,20,21] as below:

MGD = K × g× s × c

where:

K is the ESAK.

g is the g factor, which absorbs radiation energy in the breast’s glandular tissue.

s is a correction factor X-ray spectrum variation due to anode filter combinations.

c is a factor used to adjust variation in breast composition.

For DRLs, the AGD of two images is summed up and divided by two for each view to get the mean image AGD per view (AGD/view). Next, the mean image AGD/view of CC and MLO are summed up and divided by two to get the median image AGD. To determine the DRLs values, the 75th and 95th percentiles were calculated across the median image MGD for each breast thickness range.

The CBT was divided into three groups (20–39 mm, 40–59 mm, and 60–99 mm) based on the distribution of CBT in the histogram. The first group consisted of thinner breasts women whose CBT range falls between 20 and 39 mm. Women with medium breast thickness were in the second group, ranging from 40 to 59 mm. The last group had a range of 60 to 99 mm and was known as thicker breasts—the target/filter combination used for each CBT range and mammographic technique is described in Table 1.

Data were entered manually into Statistical Package for the Social Sciences (SPSS) software version 20. Descriptive statistics are used to acquire mean, median, standard deviation, percentiles, and maximum and minimum values of AGD and CBT. Normality testing was conducted using objective and subjective means, subjectively by visual observation using histograms, and objectively using Kolmogorov Smirnov (KS) test before inferential statistics. The 75th percentile values were obtained for the AGD of each mammography projection. The values obtained were tabulated separately against three CBTs for FFDM and DBT. Mann–Whitney U test was used to compare AGDs of FFDM and DBT, as the normality test for this data shows no normal distribution. The differences between AGDs across different CBT ranges were tested using Welch and Brown-Forsythe test, as the data were normally distributed, but the homogeneity of variance was violated.

## 3. Results

Table 2 shows the patients’ age for each mammographic technique. The study involved mammography patients aged between 25 and 93 years for FFDM [mean = 56.6 years; standard deviation = 10.96], and 34 to 93 years for DBT [mean = 55.0 years; standard deviation = 10.51]. Mean CBTs and exposure parameters for each mammographic technique according to the CBT range are presented in Table 3, with the mean kVps for mammographic techniques increased with increasing the CBT. The mean (range) kVp of CC and MLO for FFDM were 28.9 (26 to 33) and 29.6 (26 to 33.5), respectively. For DBT, the mean (range) kVp of CC and MLO were 31.1 (26 to 37) and 32.6 (26 to 42), respectively.

The mean, median, minimum and maximum values for AGD, CBT, ESD, and kVp of RCC, LCC, RMLO, and LMLO are presented for each mammographic technique in Table 4. The highest mean AGD was found in LMLO for FFDM [1.97 ± 1.05 mGy] and DBT [2.20 ± 0.74 mGy] while RCC recorded the lowest AGD in FFDM [1.50 ± 0.67 mGy] and DBT [1.76 ± 0.47 mGy]. The same trend was observed in ESD, with the highest ESD value recorded in LMLO for FFDM [8.23 ± 5.32 mGy] and DBT [7.34 ± 3.12]. At the same time, the lowest ESD value was observed in RCC for FFDM [5.70 ± 3.38] and DBT [5.51 ± 2.05]. The mean kVp values in all four views of FFDM were lower (ranging from 28.94–29.74) than in DBT (ranging from 31.04 ± 1.99 kVp–32.72 ± 3.02 kVp).

**Figure 1:**
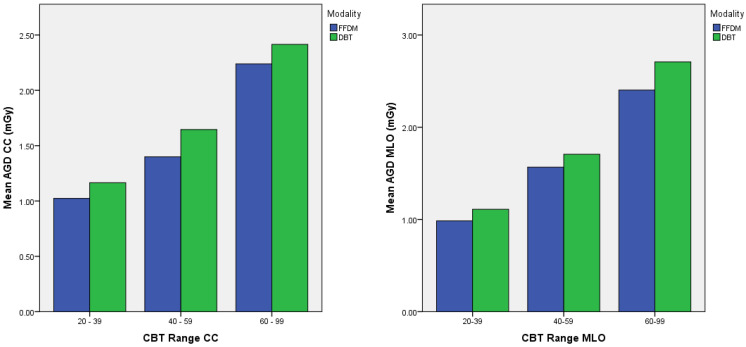
Histogram of AGD of full-field digital mammography (FFDM) and digital breast tomosynthesis (DBT) across different CBT ranges for CC and MLO projections.

Table 5 shows the mean, median, range, 75th, and 95th percentiles of AGD for all mammographic projections in FFDM and DBT. A Mann–Whitney U test indicated that the AGD of DBT was significantly higher than the AGD of FFDM for CC and MLO projections (*p* < 0.005), with the mean AGD of FFDM [CC: 1.53 ± 0.58 mGy, MLO: 1.92 ± 0.88 mGy] and DBT [CC: 1.79 ± 0.46 mGy, MLO: 2.17 ± 0.69 mGy]. The 75th percentile of AGD for CC and MLO in FFDM was 1.68 mGy and 2.25 mGy, while in DBT were 2.06 mGy and 2.59 mGy, respectively. Figure 1 shows a histogram of the AGD of FFDM and DBT across different CBT ranges for CC and MLO projections. AGD of DBT predominated over FFDM for all CBT ranges and mammographic projections.

**Figure 2:**
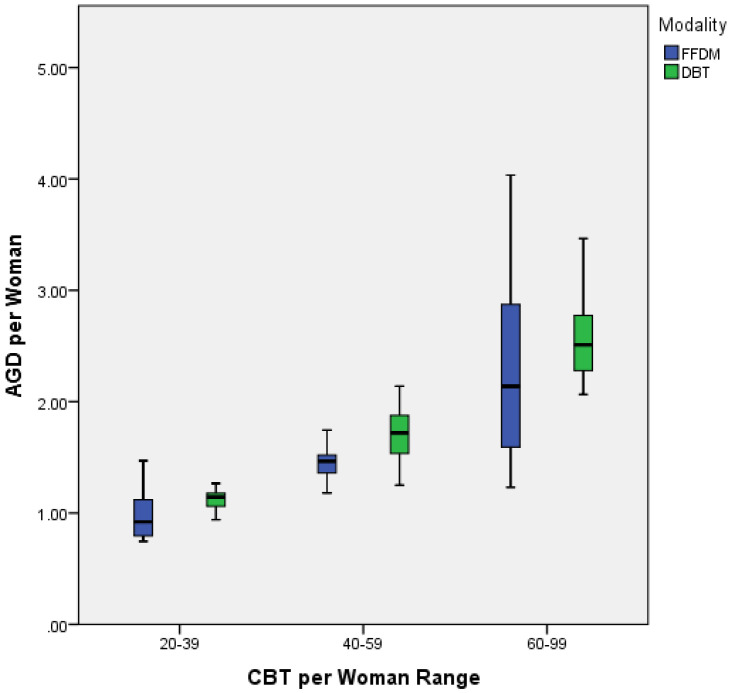
The AGD per woman at three compressed breast thickness (CBT) groups.

Table 6 shows the mean, median, 75th percentile, and 95th percentile of AGD per woman for each mammographic technique in different CBT groups. The 75th percentile was set as DRLs for each CBTs group for FFDM and DBT. Figure 2 shows an increasing trend of AGD with increasing CBT value. In FFDM, two CBT groups (20–39 mm and 40–59 mm) showed a normal distribution, and one group (60–99 mm) showed abnormal distribution. Whereas in DBT, all three groups showed normal distribution. The homogeneity of variances was violated for both FFDM and DBT groups. Both Welch and Brown-Forsythe tests indicated a significant difference across the three CBT groups for FFDM and DBT (*p* < 0.005). A Games-Howell post hoc test showed significance mean differences across all CBT groups in FFDM (*p* < 0.05) and DBT (*p* < 0.05).

The 75th and 95th percentile of AGD values of FFDM found in this study were compared with other published studies and tabulated in Table 7. All these studies determined local or institutional DRLs in their respective countries, with our local DRls of all projections in the current study demonstrated higher than other countries (2.05 mGy vs. 1.44 mGy [22] vs. 1.21 mGy [23] vs. 1.90 mGy [24]).

## 4. Discussion

This study compared local DRLs of FFDM and DBT across different mammographic techniques and compressed breast thicknesses. The mean age (55.8 ± 11.24) of the patients for FFDM and (55 ± 10.56) for DBT recorded in this study was slightly lower than the mean ages of other similar studies [(56 ± 10.0), (60 ± 7.9 years)] [22,23]. This could be due to women in Malaysia starting their screening for breast cancer at an earlier age (50 to 75 years old) compared to other countries. Additionally, the inclusion of younger women starting from 23 years old was also attributed to the lower mean age in this study because the breast cancer incidence in Malaysia is observed to rise at the age of 25 and peaks between 60 and 64 [25].

In this study, average CBTs for each mammographic technique were higher in the MLO than in the CC projection, which is in line with other studies [25,26,27]. This is mainly due to the inclusion of pectoral muscles in MLO projection that are usually thicker and denser than breast tissues.

We also found that the AGD of DBT was significantly higher than in FFDM. This finding agrees with previous studies by Svahn et al. (2015) and Ritlumlert et al. (2020) [26,27]. A study by Gennaro et al. (2018) concluded that the average increase of DBT dose compared to FFDM was 38%, and a range between 0 % and 75% [7]. Although another study by Teoh et al. (2021) found dose in DBT was slightly higher than in FFDM, in CC projection, AGD of FFDM showed somewhat higher than DBT [3.37 mGy vs. 1.86 mGy] and vice versa in the MLO view [1.37 mGy vs. 1.88 mGy] [28]. The high dose observed in DBT compared to FFDM is primarily due to the technology of DBT. DBT takes a series of 2D low-dose X-ray images from different angles and uses them to synthesize 3D images of the breast [4]. These series of exposures add up to the cumulative dose of DBT.

In this study, patients with thin breasts (less than 50 mm in thickness), who accounted for around 30% of the study population, received similar radiation doses from both mammographic techniques, FFDM and DBT. While for breast thickness more than 50 mm, the AGD of DBT was slightly lower than the dose received when using the FFDM technique. The slight difference in dose observed in both techniques is probably due to the dose reduction achieved in the DBT technique, which is partially attributed to the usage of the step and shoot technique and anti-scatter grid. The effect of the anti-scatter grid is more pronounced when used against patients with relatively thicker breast because thicker breast contributes high scatter radiation than thinner breast. A clinical study carried out in Kuwait by using the same mammography vendor (GE Senographe Essential) demonstrated a similar finding where a single-view DBT (1.8 to 4.0 mGy) yielded lower AGD than the two-view FFDM technique (3.3 to 6.0 mGy) [8]. Furthermore, 84% of the patient population in the Kuwait study comprises of 4–7 cm thickness, which is considered thicker breast owing to the lower dose of DBT than FFDM.

Furthermore, AGD has shown an increasing trend with increasing CBT. When CBT is reduced, lesser energy of radiation is used to penetrate the breast tissue, resulting in a lower AGD. Higher radiation dosage is needed to penetrate the breast tissue when CBT is increased. There was a significant difference between the AGD in FFDM and DBT across all CBT groups, in line with Suleiman et al.’s study [29]. This proves the idea that a stratified DRL should be set according to different CBTs ranges. According to International Commission on Radiological Protection (ICRP), DRL can be set for a single standard breast thickness or a range of breast thicknesses. Although selecting a particular thickness to indicate an entire community is a straightforward technique, it is less likely accurate because the population is not homogeneous, and breast thickness can range from 1 cm to 10 cm [29]. Determining DRL values for varied breast thicknesses is a highly complicated but ultimately more effective technique to improve mammography’s DRL procedure [5,27]. Moreover, breast thickness varies across different regions of the world. Women in Asia-Pacific have thinner and denser breasts than those in Europe and North America. At the same time, women in North America have substantially thicker and denser breasts on average compared to women in Europe [30]. This variation in breast thickness is inevitable due to the characteristic of geographical differences.

The comparison of DRL becomes arduous when involving different CBT ranges. In this study, DRLs set for FFDM were 1.13 mGy, 1.52 mGy, and 2.87 mGy, while DBT were 1.18 mGy, 1.88 mGy, and 2.78 mGy for CBT ranges of 20–39 mm, 40–59 mm and 60–99 mm respectively. Previously, the Malaysian government has set the national DRL for three sets of CBT ranges using the 75th percentile and patient-based method, as shown in Table 8 [17]. The national DRLs cannot be compared directly with the present study as they involve CBT changes. The CBT range set for the population involved in this study was different from the CBT used in the 2013 national DRL. The only CBT range of the current study that was similar to the national CBT range was 20–39 mm. When comparing DRLs under this particular CBT range, we can find that both DRL of FFDM [1.13 mGy] and DBT [1.18 mGy] were lower than national DRL [1.9 mGy] as recommended by ICRP [5]. This shows the facility has a good mammography practice overall and does not record dose values that deviate far from the standard range.

For ease of comparison with other countries, the DRLs have been set for CC and MLO projections. The 75th percentile and 95th percentile of AGD of the present study for CC and MLO were comparable to the findings of the study in Ghana by Dzidzornu et al. (2021) [24]. At the same time, our study recorded higher DRLs compared with studies done in Greece and Palestine [22,23]. The higher DRLs could be due to the different methods, techniques, or equipment employed in their studies. Age also could be one of the main factors attributed to the higher DRL in our study. The study by Lekatou et al. consisted of Greece women who were 40–80 years old [22], while the study by Karsh et al. consisted of Palestinian women who were 40–64 years old [23]. Still, our study consisted of women aged 25 to 93 years which showed higher involvement of the younger population. Due to more glandular and fibrous connective tissues in younger women’s breasts, a higher dose is needed to penetrate the breast for better visualization. As the results in this study were relative to other countries and the existing national DRLs, these DRLs could be seen as a preliminary to establishing national DRLs for current mammography practice in Malaysia.

There are several limitations to our study. The data is from a single institution with a relatively smaller sample size. There is also a lack of literature on stratified DRLs for different CBT ranges for DBT to compare, as presented in the current study. Besides that, we also lack data on the image quality of this study to justify the DRLs presented. Therefore, it is recommended for future studies to include a larger sample size, setting of stratified DRLs, and the inclusion of image quality data. The different protocols in setting DRLs also be considered for international comparisons.

## 5. Conclusions

In conclusion, the local DRLs in mammography from this study were lower than the national DRLs and, relative to the international standards, as well as in other countries with the AGD values of DBT were significantly higher than FFDM. Stratified DRLs according to different CBT ranges and techniques could further optimize the radiation dose delivered to the patient in mammography. Findings from this study could help future studies towards establishing national DRLs for DBT to guide practice in Malaysia. This guideline will improve the overall quality of mammographic practice in Malaysia, reducing the patient’s risk by avoiding unnecessary radiation.

## Figures and Tables

**Table 1:** The target/filter combination for each breast thickness range and mammographic technique.

CBT Range	Target/Filter Combination	
	FFDM	DBT
20–39	Molybdenum/Rhodium Molybdenum/Molybdenum Tungsten/Rhodium	Tungsten/Aluminium
40–59	Molybdenum/Rhodium Rhodium/Rhodium Tungsten/Rhodium Tungsten/Silver	
60–99	Rhodium/Rhodium Tungsten/Rhodium Tungsten/Silver	

**Table 2:** Mean and range of patients’ age involved in this study.

Modality	Age (Years)	
	Mean ± SD	Range
**FFDM**	56.55 ± 10.96	25–93
**DBT**	54.96 ± 10.51	34–93

**Table 3:** Compressed breast thickness (CBT) range and exposure parameters used in full-field digital mammography (FFDM) and digital breast tomosynthesis (DBT).

Modality	CBT Range	Number of Images	Projection	Mean CBT ± SD (mm)	Mean kVp ± SD	kVp Range
**FFDM**	20–39	17	CC	33.44 ± 4.76	26.82 ± 0.66	26.0–28.5
		13	MLO	33.65 ± 4.47	26.62 ± 0.51	26.0–27.5
	40–59	49	CC	50.77 ± 4.66	28.91 ± 0.67	28.0–30.5
		28	MLO	52.14 ± 5.38	29.18 ± 0.88	27.5–31.5
	60–99	21	CC	65.50 ± 4.86	30.76 ± 0.96	29.0–33.0
		46	MLO	70.25 ± 7.89	30.78 ± 1.21	29.0–33.5
**DBT**	20–39	22	CC	33.07 ± 5.59	28.09 ± 1.10	26.0–30.0
		17	MLO	31.91 ± 5.08	27.91 ± 0.94	26.0–29.0
	40–59	146	CC	50.10 ± 4.91	30.66 ± 0.96	29.0–32.5
		93	MLO	51.26 ± 5.12	30.90 ± 1.04	29.0–32.5
	60–99	55	CC	64.80 ± 5.12	33.58 ± 1.12	32.5–37.0
		113	MLO	69.49 ± 8.01	34.66 ± 2.13	31.5–42.0

**Table 4:** The mean, median, minimum and maximum values for AGD, CBT, ESD, and kVp for all mammographic projections in full-field digital mammography (FFDM) and digital breast tomosynthesis (DBT).

Modality	FFDM				DBT			
View	RCC	LCC	RMLO	LMLO	RCC	LCC	RMLO	LMLO
**Number of images**	87				223			
**Mean AGD ± SD (mGy)**	1.50 ± 0.67	1.55 ± 0.72	1.87 ± 0.92	1.97 ± 1.05	1.76 ± 0.47	1.81 ± 0.52	2.13 ± 0.71	2.20 ± 0.74
**Median AGD (mGy)**	1.38	1.36	1.63	1.66	1.70	1.74	2.03	2.03
**AGD range (mGy)**	0.46–5.34	0.72–4.53	0.69–6.82	0.72–6.22	0.88–4.22	0.93–4.13	0.96–5.13	0.95–4.15
**Mean CBT ± SD (mm)**	50.53 ± 12.29	51.35 ± 12.11	58.51 ± 15.29	59.40 ± 15.74	51.54 ± 10.49	52.55 ± 10.82	58.48 ± 13.95	59.57 ± 14.02
**CBT range (mm)**	22–87	21–81	24–99	25–92	20–87	22–81	23–114	23–93
**Mean ESD ± SD (mGy)**	5.70 ± 3.38	5.98 ± 3.42	7.64 ± 4.55	8.23 ± 5.32	5.51 ± 2.05	5.67 ± 2.22	7.05 ± 3.00	7.34 ± 3.12
**ESD range (mGy)**	0.97–26.05	1.68–21.12	1.74–32.26	1.91–31.65	1.65–15.59	1.37–15.33	1.91–18.34	1.91–15.64
**Mean kVp ± SD**	28.94 ± 1.62	28.95 ± 1.54	29.55 ± 1.87	29.74 ± 1.89	31.04 ± 1.99	31.22 ± 2.02	32.44 ± 2.87	32.72 ± 3.02
**kVp range**	25–33	26–33	26–36	26–34	26–40	26–38	26–46	26–42

AGD, average glandular dose; CBT, compressed breast thickness; ESD: entrance surface dose; kVp, kilovoltage peak; RCC, right craniocaudal; LCC, left craniocaudal; RMLO, right mediolateral oblique; LMLO, left mediolateral oblique; SD, standard deviation; mGy, milligray.

**Table 5:** Mean, median, range, 75th, and 95th percentiles of AGD for all mammographic projections in full-field digital mammography (FFDM) and digital breast tomosynthesis (DBT).

Projections	Modality	AGD (mGy)					
		Mean ± SD	Mann–Whitney U Test	Median	Range	75th Percentile	95th Percentile
**CC**	FFDM	1.53 ± 0.58	*p* < 0.05	1.40	0.59–3.53	1.68	2.92
	DBT	1.79 ± 0.46		1.69	0.92–3.45	2.06	2.68
**MLO**	FFDM	1.92 ± 0.88	*p* < 0.05	1.65	0.71–4.54	2.25	3.94
	DBT	2.17 ± 0.69		2.08	0.96–4.28	2.59	3.53

**Table 6:** The mean, median, 75th percentile, and 95th percentile of AGD per woman for each mammographic technique in different CBT groups.

Modality	FFDM			DBT		
**CBT Range**	20–39	40–59	60–99	20–39	40–59	60–99
**Number of images**	13	37	37	16	125	82
**Mean AGD ± SD (mGy)**	0.97 ± 0.22	1.46 ± 0.13	2.26 ± 0.76	1.11 ± 0.09	1.70 ± 0.22	2.57 ± 0.37
**Median AGD (mGy)**	0.92	1.47	2.13	1.14	1.72	2.51
**75th percentile of AGD (mGy)**	1.13	1.52	2.87	1.18	1.88	2.78
**95th percentile of AGD (mGy)**	-	1.70	3.67	-	2.03	3.35
**Welch and Brown-Forsythe tests**	*p* < 0.005			*p* < 0.005		

**Table 7:** Comparison of the compressed breast thickness (CBT) and AGD values of full-field digital mammography (FFDM) at 75th and 95th percentile with corresponding previously published values.

Author(s) (Year)	Number of Women	CBT (mm)	AGD (mGy)		
			Mean	75th Percentile	95th Percentile
**Lekatou, Metaxas** [22]	300	All: 56.3 CC: 53.9 MLO: 58.6	All: 1.25 CC: 1.18 MLO: 1.32	All: 1.44 CC: 1.41 MLO: 1.48	All: 1.77 CC: 1.76 MLO: 1.78
**Karsh** [23]	200	All: 61.32 CC: 56.92 MLO: 66.5	All: 1.06 CC: 0.98 MLO: 1.13	All: 1.21 CC: 1.12 MLO: 1.28	All: 1.56 CC: 1.46 MLO: 1.64
**Dzidzornu, Angmorterh** [24]	979	All: 40 CC: 36 MLO: 45	All: 1.8 CC: 1.6 MLO: 2.0	All: 1.9 CC: 1.6 MLO: 2.4	All: 4.1 CC: 3.0 MLO: 4.6
**Current study**	87	All: 55.19 CC: 50.94 MLO: 58.95	All: 1.73 CC: 1.53 MLO: 1.92	All: 2.05 CC: 1.68 MLO: 2.25	All: 3.45 CC: 2.92 MLO: 3.94

**Table 8:** Diagnostic Reference Level for mammography in Malaysia [17].

	Breast Thickness (cm)	DRLs in MGD (mGy)
Ministry of Health (2013) [17]	2–3.9	1.9
	4–7.9	2.0
	8–10	3.2

## Data Availability

The data presented in this study are available on request from the corresponding author. The data are not publicly available for ethical purposes.
